# Case report and presentation of a new classification system for hip acetabular and periacetabular ossifications and calcifications

**DOI:** 10.4102/sajr.v28i1.2874

**Published:** 2024-05-31

**Authors:** Luis Perez-Carro, Oscar Perez-Fernandez, Alvaro Cerezal Canga, T Jegathesan, Luis Antonio Ruiz-Villanueva, Luis Cerezal-Pesquera

**Affiliations:** 1 Department of Orthopaedic Surgery, Hospital Mompia, Santander, Spain; 2 Department of Orthopaedic Surgery, Hospital La Paz, Madrid, Spain; 3 Department of Orthopaedic Surgery, Hospital Tan Tock Seng, Singapore; 4 Department of Radiology, Diagnostico Medico Cantabria, Santander, Spain

**Keywords:** hip, arthroscopy, labrum, rectus femoris, ossification

## Abstract

This report describes a rare case in which double calcifications of the acetabular labrum and rectus femoris occurred concomitantly in a middle-aged female patient who was treated successfully with surgical intervention via hip arthroscopy.

**Contribution:**

This case highlights the existence of various types of calcifications around the acetabulum, with a proposed new classification system for acetabular and periacetabular rim ossifications.

## Introduction

Calcifications are well-established pathophysiologic entities that are present in muscles, tendons and ligaments throughout the body and have been described as chondrocyte-mediated responses to injury or hypoxia.^1^ With the advancement of hip arthroscopy, studies on the acetabular labrum and pathology have taken greater interest. To date, there are a handful of review articles on calcifications of the acetabular labrum and scattered case reports on calcific tendinitis of the rectus femoris.

A middle-aged woman presented with hip pain and calcification in both the labrum and rectus femoris. She subsequently underwent surgery, which eventually resolved her symptoms. To the best of the authors’ knowledge, there have been no reports of calcifications occurring simultaneously in both the hip labrum and the rectus femoris.

## Case report

A 42-year-old woman with no prior medical history or trauma presented with a 2-year history of bilateral hip pain, worse on the right. The site of pain was mainly located in the gluteal region, groin and sacrum. The pain was mechanical in nature and aggravated by activity and hip flexion. Prior to her being admitted to our specialist clinic, she had undergone oral analgesia and multiple regional injections (sacroiliac and trochanteric), which provided only temporary relief. The patient had a normal body mass index and a normal gait. Passive deep hip flexion was painful, as was the flexion, adduction, and internal rotation (FADDIR) manoeuvre. The flexion, abduction and external rotation (FABER) test was negative. Passive flexion-extension and internal and external rotation of her hip were within the normal range, and there was no abnormality in her lower limb rotational profile or limb length. Her modified Harris hip score (MHHS) was 63, her HOS-ADL (Hip Outcome Score with Activities of Dailing Living Subscale) was 73, and her HOS-SS (Hip Outcome Score with Sports-Specific Subscale) was 46.

**FIGURE 1:**
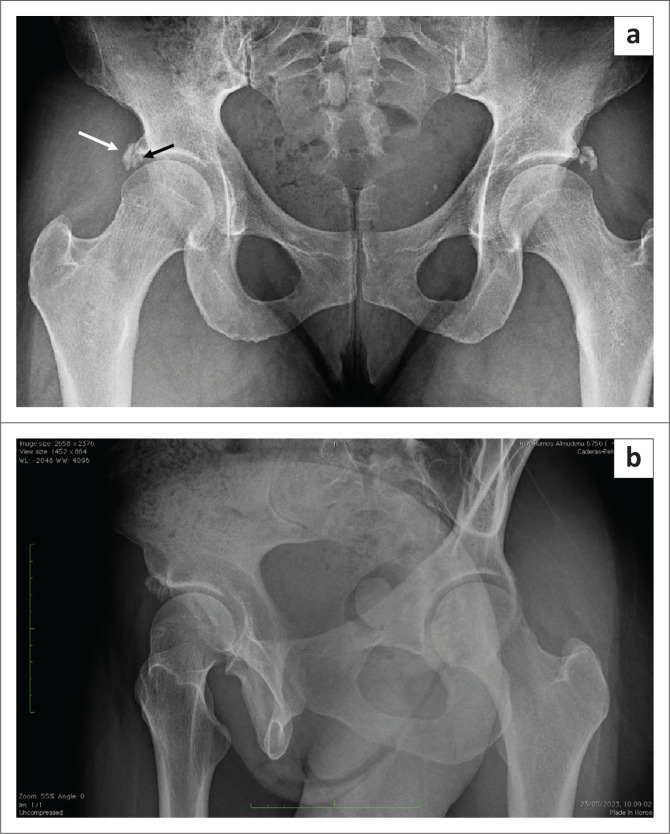
(a) Anteroposterior pelvis radiograph. Double calcifications are seen pre-operatively at the superolateral edge of both acetabula, separated by a subtle radiolucent line and a small subarticular cyst. The calcification of the rectus femoris originating from the anterior inferior iliac spine is denoted on the right hip by the solid white arrow, while the punctate calcifications of the acetabular labrum is denoted by the solid black arrow. (b) C Profile view revealing the labral and the rectus calcifications, a synovial herniation pit and the cam morphology of the femoral head.

Plain radiographs of her pelvis demonstrated calcifications around the superolateral aspects of both acetabula, a synovial herniation pit, cam morphology and a small subarticular cyst (Figure 1a and Figure 1b). Upon scrutiny, the calcifications were seen arising from two distinct origins. The double calcifications were separated by a subtle radiolucent line, with the calcification of the rectus femoris being round and originating from the anterior inferior iliac spine, while punctate and amorphous calcifications of the acetabular labrum were observed to arise beneath the superolateral rim of the right acetabulum. Similar calcifications were also observed on the contralateral left hip. For other relevant hip imaging parameters, the lateral centre edge angle of the right hip was 30°, the alpha angle was 69°, the Tonnis angle was 8°, and the articular space was normal. Advanced imaging was obtained via MR arthrogram of the right hip, revealing rupture of the anterior-superior labrum with early chondral delamination. The patient was informed of her diagnosis of right hip femoral acetabular impingement (FAI) with chondrolabral rupture and rim calcifications and was advised on surgical intervention in the form of right hip arthroscopy.

**FIGURE 2:**
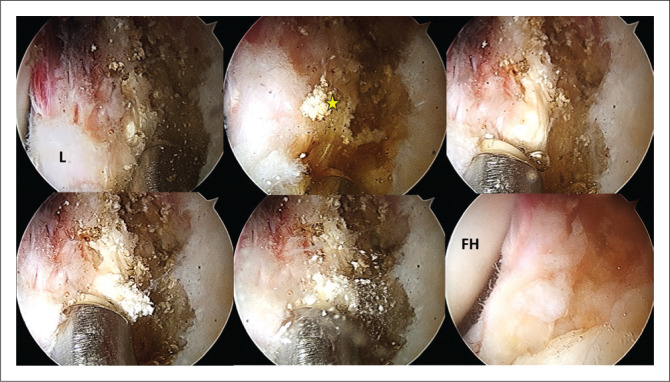
Right hip. Arthroscopy images demonstrating the labral calcifications (yellow star). They were expressed, debrided, and evacuated with radiofrequency and a shaver device. Bottom right: Labrum post removal of calcification. L (labrum) FH (femoral head).

**FIGURE 3:**
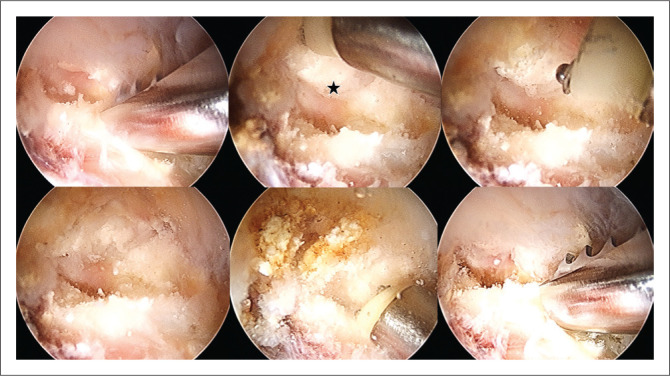
Right hip. Arthroscopy images showing the rectus (direct head) calcifications (Black star) and the removal process.

Hip arthroscopy was performed under combined anaesthesia (spinal and general) in the supine position with standard anterolateral (AL), modified anteromedial (MAP) and distal anterolateral (DALA) portals. Interportal capsulotomy was performed to improve central compartment access. Diagnostic arthroscopy revealed a tear of the superior and anterosuperior labrum from 12:00 to 14:00. During probe mobilisation of the ruptured labrum, calcifications were identified anterosuperiorly within the labrum and the capsule-labral recess (Figure 2). During decompression of the subspine region, calcifications were also found within the rectus femoris (direct portion) (Figure 3). These calcifications were easily expressed, debrided and evacuated with radiofrecuency and a shaver device without resection of any labral tissue. It was impossible to retrieve samples from these calcifications because of their punctate nature so no histopathological analysis was performed. The ruptured anterior labrum was repaired with three Iconix (Stryker®) suture anchors. A T-shaped capsulotomy was performed for better visualisation of the femoral neck. There was a large anterolateral cam lesion that underwent femoral osteoplasty with a burr under fluoroscopic guidance. Total capsular closure was performed at the end of surgery.

**FIGURE 4:**
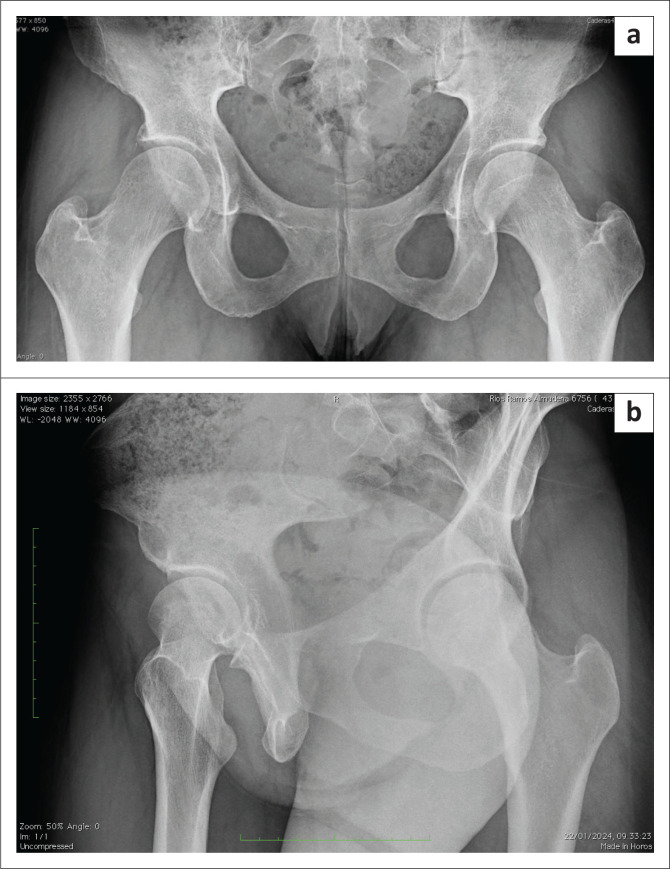
(a) Post-operative plain anteroposterior pelvis radiograph showing almost complete resolution of the calcifications at both hip joints after right hip arthroscopy with residual faint linear calcifications. (b) Right hip. Profile radiograph showing complete removal of the calcifications and cam excision.

The patient was discharged the next day and adhered to the standard postoperative recovery process for labral repair and osteochondroplasty. This involved using crutches for 3 weeks, performing circumduction movements, and using a stationary bicycle for rehabilitation. The patient had no postoperative complications and received heterotopic ossification prophylaxis (Naproxen 500 mg twice daily) after the procedure for 3 weeks. At the outpatient follow-up visit 3 months after surgery, plain radiographs (Figure 4a and b) revealed almost complete disappearance of the previously observed calcifications in the right hip and surprisingly, spontaneous resolution in the untreated left hip with faint linear residual calcification bilaterally. At the 1-year follow-up, there was no recurrence of the ossifications, and the symptoms had completely resolved. Her MHHS was 92, her HOS-ADL score was 93, and her HOS-SS score was 92.

## Discussion

Calcifications around the acetabular rim have been reported in the literature, and several well-defined forms of these calcifications have been described, including labral calcifications, stress fractures of the acetabular rim, apophysitis of the acetabular rim and persistent secondary ossification centres of the acetabulum.^2^ Mineralisation may be because of calcification or ossification. Strictly speaking, calcification and ossification are different entities. Ossification implies new bone formation, whereas calcification is the process in which calcium salts build up in the soft tissue. Although their pathophysiology is poorly understood, they are mostly linked to femoroacetabular impingement.^2^ The presence of labral calcifications has been shown to be associated with ongoing synovial damage, with preclinical data also demonstrating that calcium crystals can activate intra-articular proinflammatory pathways and release nociceptor stimulating substances. Thus, calcium crystal deposition may be involved in generating pain within and around the hip joint.^3^

The labral calcification described in this case was punctate and amorphous in nature, and the removal of calcium deposits left sufficient high-quality residual labral tissue for repair. This is in contrast to the completely calcified labral or os acetabuli. In a recent study, Marc Safran^2^ provided a framework to better identify and categorise acetabular rim ossifications variants that are found in almost 20% of patients presenting with hip pain. These lesions were divided based on their morphological appearance: (1) punctate or partial calcifications within the labrum; (2) large, rounded calcifications (true os acetabuli); (3) large fragments with a vertical line on the superior-lateral acetabular rim consistent with healed or non-healed stress fractures; and (4) complete circumferential ossification of the labrum, contiguous with the lateral edge of the acetabular rim. In the setting of FAI, it has been postulated that persistent impingement of morphologically abnormal acetabula or proximal femora may instigate the production of calcifications between the native acetabular rim and labrum or within the labral substance itself.^4^

The rectus insertion has been described as a potential site of calcification and pain within the joint. These lesions are often extra-articular on radiography, and they represent a separate pathology to the entities described above as acetabular rim ossifications. Traumatic, genetic and local metabolic factors have been previously postulated as possible mechanisms of calcific tendinitis of the rectus femoris.^5^ The reflected head that originates at the ilium above the acetabulum is more commonly affected by calcific tendinitis than the straight head.^6^ Although most cases of calcific tendinitis are self-limiting, patients with moderate to severe discomfort can benefit from treatment options, including endoscopic or arthroscopic excision.^7^ As a result of the presence of cam deformity in the presented case, ultrasound guided barbotage or lavage and steroid injection were not considered. Recent improvements in hip arthroscopy have allowed for treatment of both intra-articular and extra-articular disorders, with good recovery.

Heterotopic ossification (HO) post-hip arthroscopy is a known complication that is not uncommon and is another possibility for acetabular and periacetabular ossifications.^8,9^ Surgical intervention in the form of hip arthroscopy can be considered for patients with symptoms refractory to conservative management because of the good resolution of both symptoms and calcifications. Notably, in this patient, calcifications on the contralateral hip also disappeared spontaneously after the surgical intervention on the right hip. The authors of this report postulate that this was likely because of the combination of reduced mobilisation postoperatively, as well as the chemoprophylaxis taken to protect against HO, which may have helped with the clearance of calcifications in the contralateral hip.

**FIGURE 5:**
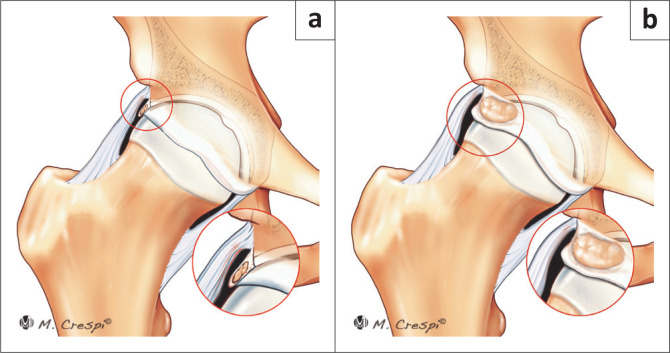
New classification system for patients with acetabular and periacetabular rim ossifications. (a) Type A1: Punctate and or partial calcifications within the labrum. (b) Type A2: Large, rounded calcifications (true os acetabuli).

**FIGURE 6:**
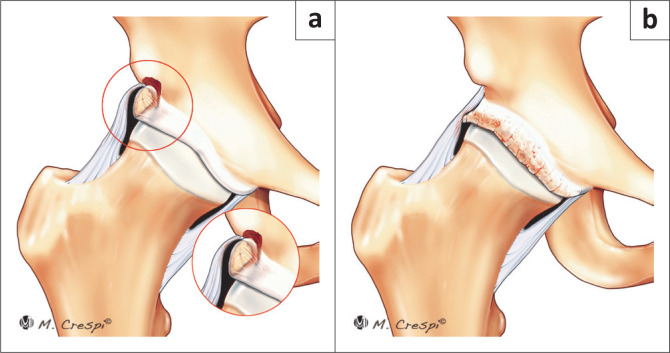
New classification system for patients with acetabular and periacetabular rim ossifications. (a) Type A3: Large fragments with a vertical line on the superior-lateral acetabular rim consistent with healed or non-healed stress fractures. (b) Type A4: Complete circumferential ossification of the labrum contiguous with the lateral edge of the acetabular rim.

**FIGURE 7:**
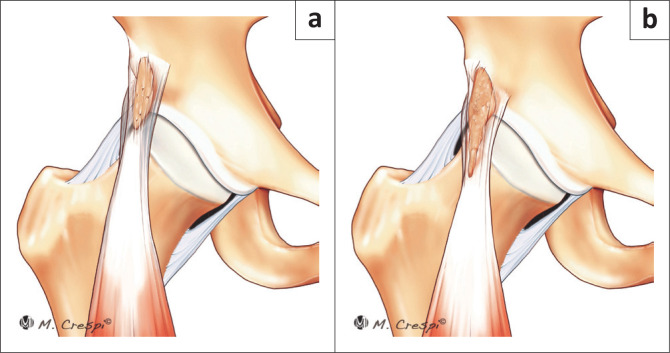
New classification system for patients with acetabular and periacetabular rim ossifications. (a) Type B1: Rectus direct head ossification: Filiform short. (b) Type B2: Rectus direct head ossification: Filiform long.

**FIGURE 8:**
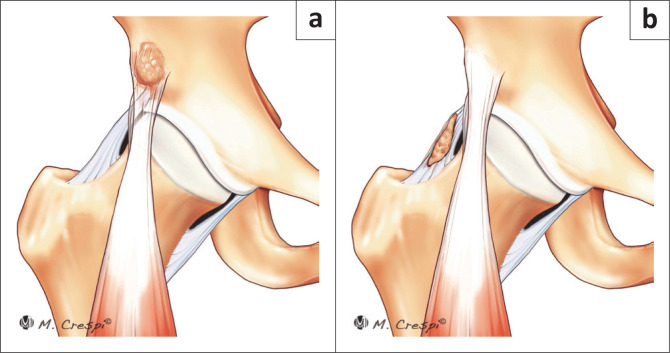
New classification system for patients with acetabular and periacetabular rim ossifications. (a) Type B3: Rectus direct head ossification: Rounded or diffuse. (b) Type C: Heterotopic capsular ossification.

This case report highlights the existence of various types of calcifications and ossifications around the acetabulum, and we propose a new classification system for patients with acetabular and periacetabular rim ossifications or calcifications (Table 1, Figure 5, Figure 6, Figure 7 and Figure 8). The value of plain radiographs is very important as these small areas of calcification or ossification are easily missed on MRI. Distinction between the acetabulum (bone), acetabular rim (rim) and periacetabular (at a distance from the rim) locations of the calcifications is very important for the diagnosis. The presence of small multiple punctate labral calcifications (Type A1) should alert the reporting radiologist to look for features of FAI as well as dysplasia and osteoarthritis. Os acetabuli (Type A2) represent persistent secondary ossification centres that may have been prevented from fusing by repetitive impingement and acetabular rim stress fractures, and are usually the result of chronic repetitive microtrauma. Thus, the presence of these entities should alert the radiologist to look for features of acetabular overcoverage and impingement. Os acetabuli may be bilateral, round or ovoid in shape, often corticated with a concave lateral border and convex medial border and are usually situated slightly away from the acetabulum, separated by an oblique radiolucent line. In contrast, acetabular rim stress fractures (Type A3) have a straight medial border with a vertical cleft separating them from the acetabulum. B1–B3 refer to calcific tendinitis of the direct head of the rectus but the radiologist should be aware that there are other causes of mineralisation related to the proximal rectus such as anteroinferior iliac spine avulsions or the different types of subspine impingement. Type C (heterotopic capsular ossification) represents true ossification and thus may appear more dense and corticated on imaging. There are four types of HO as described by Brooker.^9^ Type 1: Islands of bone within the soft tissue about the hip. Type 2: Bone spurs from the pelvis or proximal end of the femur, leaving at least 1 cm between the opposing bone surfaces. Type III: Bone spurs from the pelvis or proximal end of the femur, reducing the space between opposing bone surfaces to less than 1 cm. Type IV: Apparent bone ankylosis of the hip.

**TABLE 1:** Types of acetabular and periacetabular rim ossifications and calcifications.

**Type A: Acetabular rim ossifications/calcifications (Safran’s classification^2^)** A1: Punctate/partial calcifications within the labrum. A2: Large, rounded calcifications (true os acetabuli). A3: Large fragments with a vertical line on the superior-lateral acetabular rim consistent with healed or non-healed stress fractures. A4: Complete circumferential ossification of the labrum contiguous with the lateral edge of the acetabular rim. **Type B: Rectus direct head calcification** B1: Filiform short B2: Filiform long B3: Rounded or diffuse **Type C: Heterotopic capsular ossification (Brooker’s classification of heterotopic ossification^9^)** **Type D: Combination of A and B: Rim and rectus.** Double layer periacetabular rim calcification

## Conclusion

This case report draws attention to the existence of various types of calcifications around the acetabulum. The role of periacetabular calcifications in the clinical evaluation and outcomes of patients with femoroacetabular impingement requires further investigation, and this new classification of acetabular and periacetabular rim ossifications may be helpful.
